# The Scavenger Receptor SREC-I Cooperates with Toll-Like Receptors to Trigger Inflammatory Innate Immune Responses

**DOI:** 10.3389/fimmu.2016.00226

**Published:** 2016-06-13

**Authors:** Ayesha Murshid, Thiago J. Borges, Benjamin J. Lang, Stuart K. Calderwood

**Affiliations:** ^1^Molecular and Cellular Radiation Oncology, Center for Life Sciences, Beth Israel Deaconess Medical Center, Harvard Medical School, Boston, MA, USA; ^2^Biomedical Research Institute, School of Biosciences, Pontifícia Universidade Católica do Rio Grande do Sul (PUCRS), Porto Alegre, Brazil

**Keywords:** SREC-I, TLR3, TLR4, innate immunity, adaptive immunity

## Abstract

Scavenger receptor expressed by endothelial cell-I (SREC-I) is a class F scavenger receptor expressed by immune cells with a significant role in CD8^+^- and CD4^+^-mediated T cell immunity. This receptor can also modulate the function of toll-like receptors (TLRs), which play essential roles in innate immunity. Earlier, it was found that human monocyte/macrophage THP1 cells and bone marrow-derived macrophages from mice exhibited increased responses to polyinosine–polycytidylic acid (poly I:C, PIC) and CpG (unmethylated) DNA and enhanced production of inflammatory cytokines with overexpressed SREC-I. Our data also showed that intracellular/endocytic TLR3 and TLR9 could directly interact with SREC-I in the presence of their respective ligands. We also observed that the internalized ligand along with TLR3/TLR9 colocalized in the endosome in macrophages and THP-1 cells overexpressing these receptors. In the absence of these ligands, there was no detectable colocalization between the SREC-I and endocytic TLRs. Earlier, it was shown that SREC-I stimulated double-stranded RNA/CpGDNA-mediated TLR3/TLR9 activation of the innate immune response by triggering signaling through the NF-κB, IRF3, and MAP kinase pathways leading to transcription of cytokine genes. We also established that SREC-I can associate with plasma membrane TLRs, such as TLR2 and TLR4. We demonstrated that SREC-I–TLR4 signals more efficiently from lipid microdomain in which lipopolysaccharide (LPS) can associate with SREC-I–TLR4 complex. We also proved that SREC-I is an alternate receptor for LPS capable of internalizing the complex and for endocytic TLR ligands as well. This binding activated endocytic TLR-mediated downstream cytokine production in THP1 cells and macrophages. Finally, SREC-I could also form complexes with TLR2 and induce the release of cytokines in the presence of bacterial, viral, and fungal ligands.

## Introduction

Scavenger receptors constitute a large family of protein molecules, which were identified by Brown and Goldstein in the year 1979 (1, 2). Their function was first characterized as the receptors capable of scavenging oxidized low-density lipoprotein (ox-LDL) (3). While initially identified to recognize modified self-molecules, SRs have since been shown to also recognize numerous pathogen-derived molecules and regulate the ensuing immune response. SRs are categorized into 10 class types designated A–J, although with very little sequence conservation between these groups. Nevertheless, this apparent lack of homology between SR classes is not reflected by the number of ligands recognized by multiple SR members as many of the structurally distinct SRs recognize common ligands (4–7). The ability of SRs to transmit ligand-specific biological signals combined with various ligands bound by some individual SR members is both remarkable and yet to be fully understood at the molecular level.

Here, we discuss how a member of scavenger receptor family F, scavenger receptor expressed by endothelial cell-I (SREC-I), cooperates with toll-like receptors (TLRs) and modulates its downstream signal activation in response to specific ligand stimulation. Also known as SCARF-I, SREC-I is an 86-kDa protein with an extended extracellular domain, which is composed of epidermal growth factor (EGF)-like cysteine rich motifs, characteristic of the class F group of SRs (8–10). Known SREC-1 ligands include modified LDL (including oxidized, acetylated, and carbamylated forms), lipopolysaccharide (LPS), apoptotic bodies, Hsp70, Hsp90, calreticulin, gp96, and zymogen granule protein 2 (GP2) (11). In the case of apoptotic bodies, heat shock protein (Hsp)70, Hsp90, calreticulin, gp96, and GP2, SREC-I recognition leads to engulfment and/or endocytosis. In the absence of known ligands, SREC-1 was shown to promote cell–cell homophilic interactions between murine fibroblast cells, an effect that was amplified upon coculture with SREC-II-expressing cells and negated by the SREC-I ligands AcLDL and ox-LDL (9). These findings identified a potential ligand-independent role for SREC-I in cell–cell interactions, a function possessed by other SRs, such as LOX-1, which was shown to facilitate leukocyte–endothelium adhesion (12). SREC-I receptor has also been shown to induce morphological changes in neurons *via* its intracellular domain (4).

SREC-I was found to be a key receptor for HSPs and also to play a key role in immune response (13–15). It was demonstrated that HSP–tumor antigen complexes could be internalized after interacting with this receptor expressed in antigen-presenting cells such as dendritic cells. Antigens internalized in this way could later be processed and presented to T cells, thereby activating adaptive immunity. It was shown that HSP-chaperoned tumor antigen could be presented to both CD8^+^ and CD4^+^ T cells to activate immune response, which is also known as T cell priming (14, 15). However, further investigation is required to understand basic mechanisms and selectivity of SREC-I involved in T cell priming *via* MHCI and MHCII molecules. SREC-I, a potent antigen cross-presenting HSP receptor, is also known to induce inflammatory responses through interaction and cross-talk with another group of receptors – TLRs. One method by which microbes are detected by innate cells is through engagement of these pattern-recognition receptors (PRRs). TLRs recognize the presence of pathogen-associated molecular pattern (PAMP) molecules, leading to downstream signal transduction triggering and the activation of innate cells (16, 17). While some TLR members, such as TLR1, 2, 4, 5, and 6, can be detected on the plasma membrane and recognize components of microbial membranes, others, such as TLR3, 7, and 9, are intracellular proteins are characterized as “endosomal TLRs” and recognize nucleic acids (16, 18). In some cases, SREC-I can cooperate with TLRs in signal transduction, and this molecule was demonstrated to be important as a scavenger receptor in the control of infections (19–22). SREC-I has been shown to cooperate with multiple TLRs to transmit ligand-specific signals and to function as a potent antigen-presenting receptor for HSP-associated antigens.

## SREC-I Cooperates with TLR4 to Activate LPS-Induced Inflammation

**Figure 1:**
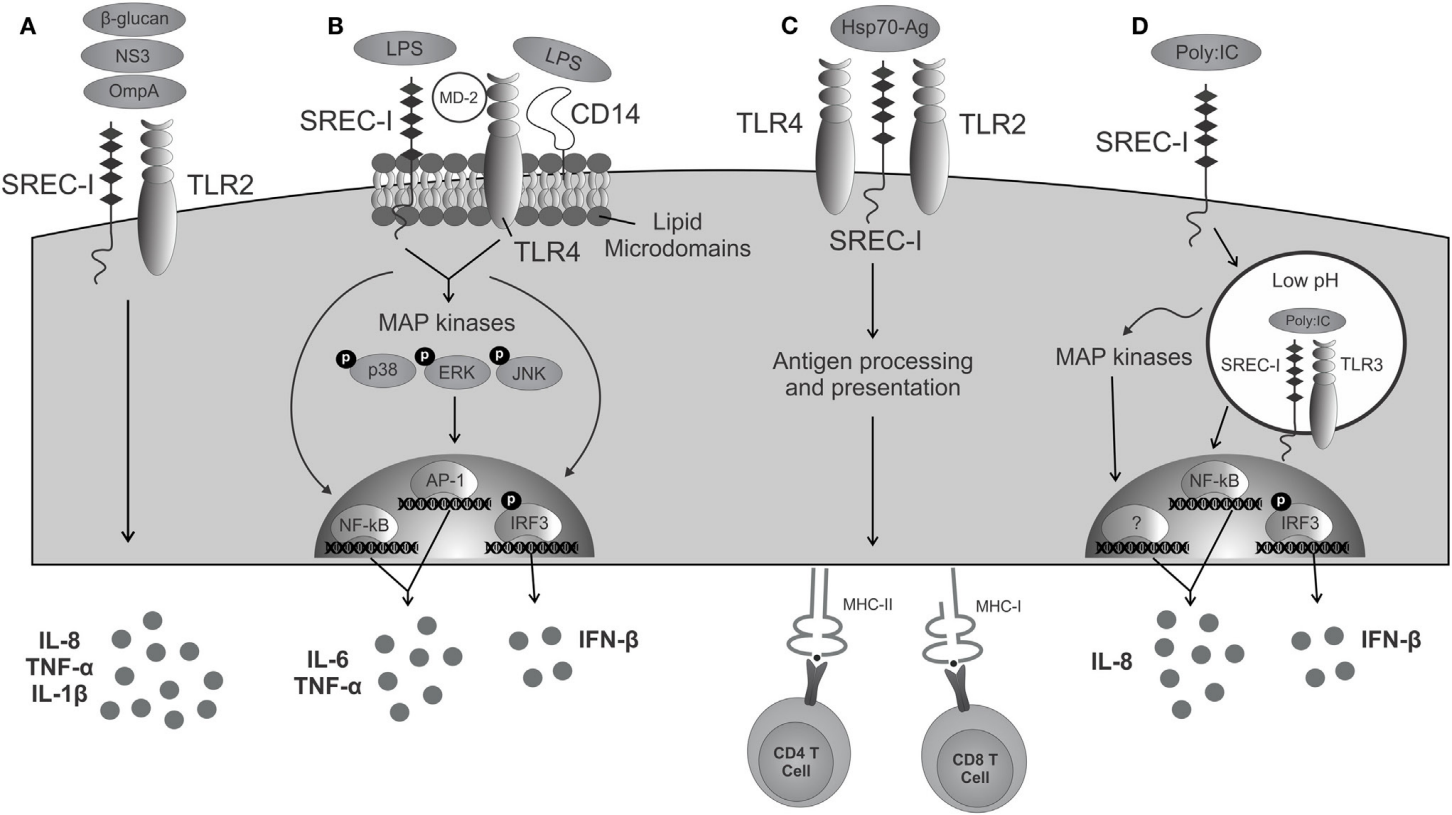
**SREC-I triggered immunity in cooperation with TLRs**. **(A)** Molecules derived from pathogens are recognized by both SREC-I and TLR2, activating signaling, and triggering the release of cytokines. **(B)** The complex LPS/SREC-I/MD-2/TLR4/CD14 launches an inflammatory cascade mediated by the activation of MAP kinases, transcription factors, and the presence of inflammatory cytokines. **(C)** Hsp70–Ag interacts with SREC-I/TLR complexes present on antigen-presenting cells and then becomes internalized. Acquired antigens are processed and presented to MHC class I and class II molecules activating CD8^+^ and CD4^+^ T cells, respectively. **(D)** SREC-I can recognize viral dsRNA such as PIC. SREC-I engagement leads to the recruitment of the complex SREC-I/TLR3/PIC to endosomes with low pH. TLR3 is then able to trigger the activation of MAP kinases, transcription factors, and the production of inflammatory cytokines.

To date, TLR4 is the most studied TLR member. TLR4 initiates inflammatory responses upon binding to LPS of Gram-negative bacteria. In this context, transduction of the TLR4 activation signal is facilitated by adaptor molecules, such as CD14 and MD-2. CD14 is termed as a recognizing receptor for TLR4. Once activated, TLR4 recruits the TIR domain-containing adaptors (TIRAP) and MyD88 and activates transcription factors, such as NF-κB, IRF3, NF-IL6, and AP-1, that will lead to the production of pro-inflammatory cytokines (23). In some circumstances, SREC-I can recognize LPS in the absence of CD14 (24) (Figure 1). Upon LPS binding, SREC-I and TLR4 activate NF-κB and MAP kinases and the subsequent production of inflammatory cytokines, such as IL-6, TNF-α, and IFN-β (25). Interestingly, IL-1α, IL-1β, and TNF-α were shown to inhibit SREC-I promoter activity, potentially representing a negative feedback loop to limit inflammatory signaling during contexts where levels of inflammatory cytokines are high (26). In the presence of LPS, SREC-I was shown to cause the translocation of TLR4 to lipid microdomains on cell surface (25) where signaling event initiates. Then, this complex (LPS–TLR4–SREC-I) appeared to become internalized to intracellular endosomes (25). In the presence of LPS, SREC-I and TLR4 translocated into lipid microdomains on plasma membrane and initiated pro-inflammatory signaling events from this location (Figure 1). We observed their presence in lipid microdomain when isolated using Optiprep density gradient centrifugation. One could hypothesize that the localization of SREC-I to lipid microdomains in the presence of some ligands promotes SREC-I interaction with certain TLRs by concentrating their proximity in these membrane substructures. Lipid microdomains are important signaling nexi for many surface molecules (27).

## SREC-I and Its Collaborative Function with TLR2

TLR2 can recognize a broad range of bacterial-, parasitic-, viral-, and fungal-derived PAMPs (28). This receptor can form heterodimers with other cell surface TLRs, such as TLR1 and TLR6, depending on its ligand (28). TLR2 also cooperates with SREC-I in the recognition of certain pathogens. For example, SREC-I and the other c-type lectin, scavenger receptor LOX-1 induced the production of IL-6 and IL-8 in the presence of outer membrane protein A (OmpA) from *Klebsiella pneumonia*, a response dependent on TLR2 (19). SREC-I also recognized β-glucans present on the cell surface of fungi species *Cryptococcus neoformans* and *Candida albicans* and triggered the production of IL-1β and the chemokines, CXCL2 and CXCL1 in association with TLR2 (20). After hepatitis C virus stimulation, dendritic cells could recognize and lead to endocytosis of the non-structural protein 3 (NS3) through SREC-I and produce IL-6 in a TLR2-dependent manner (21), although there was no evidence of their direct interaction or binding.

## Endocytic TLR3 Requires SREC-I for Ligand Recognition and Internalization in Some Monocytic Cells

During viral infections, viral double-stranded RNA (dsRNA) can be recognized by immune cell as a PAMP, indicating viral infection (29, 30). TLR3 is an intracellular member of the TLR family, present in the endosomes, and it has a unique capacity of recognizing and activation by viral dsRNA (31, 32). Once engaged by viral dsRNA, TLR3 signals through a molecular pathway that requires adaptor protein TRIF to activate transcription factors, IRF3 and NF-κB, triggering the production of type I interferon (IFN-1) and inflammatory cytokines, such as IL-8 and IL-6 (33). The EGF, ErbB1 and Btk, can phosphorylate two tyrosine residues in the cytoplasmic domain of TLR3 in order to facilitate interaction with TRIF (34, 35).

Despite most commonly being reported to reside in the intracellular endosomes, TLR3 has also been observed on the cell surface in some endothelial, epithelial, and fibroblastic cells in the presence of dsRNA and UNC93B1 (an accessory TLR protein) (36–38). The TLR3 ectodomain is required for its translocation to the plasma membrane (39). Recently, it was shown that TLR3 can interact with SREC-I in the presence of TLR3 ligand. Upon PIC (poly I:C, dsRNA) treatment, TLR3 and SREC-I can colocalize to the endosomes in THP-1 monocytes (40). The formation of the SREC-I–TLR3–PIC complex led to higher rates of NF-κB pathway activity and greater expression of phosphorylated (activated) MAP kinases p38 and c-jun kinase (JNK), along with secretion of pro-inflammatory cytokines, such as IL-8 and IL-6 (40) (Figure 1). Thus, TLR3 occupies SREC-I as a coreceptor and enhances its PIC-mediated activation (40).

## SREC-I in Immunity and Disease: What Remains Unknown and Future Directions

SREC-I has been characterized as a receptor for extracellular HSPs, gp96, and modified LDL. This promiscuous receptor is a key component of innate immunity and is capable of recognizing TLR ligands, such as LPS, unmethylated DNA, or dsRNA. However, it has recently been shown to participate both in innate and adaptive immunity in endothelial cells, fibroblasts, and immune cells. In addition to internalizing HSPs, Means’ group demonstrated that SREC-I can endocytose apoptotic cells by recognizing phosphatidylserine exposed on the outer leaflet of the plasma membrane and with the help of complement factor C1q (41). They also strongly reported that failure of this removal *in vivo* resulted in spontaneous development of autoimmune disease (41). It would be interesting to find out more how SREC-I can protect against autoimmunity and to understand the molecular basis for this property of SREC-I. Such work may ultimately enable modulation of SREC-I activity in contexts of autoimmunity for therapeutic benefit. Indeed, it has been shown that defective clearance of apoptotic cells by the scavenger receptors increases susceptibility to lupus (41). In addition to contexts of autoimmunity, understanding SREC-I function in apoptotic cell uptake and identifying its interacting partners will also open up the field to understanding infection-driven immune responses regulated by SREC-I. For its functional versatility and versatility in ligand, recognition makes it the scavenging Jack of all Trades.

## Author Contributions

AM, TJB and BJL wrote the manuscript. SKC oversaw the study and provided intellectual input. All authors approved the manuscript for publication.

## Conflict of Interest Statement

The authors declare that the research was conducted in the absence of any commercial or financial relationships that could be construed as a potential conflict of interest.
